# Networking Skills: The Effect of Graphene on the Crosslinking of Natural Rubber Nanocomposites with Sulfur and Peroxide Systems

**DOI:** 10.3390/polym14204363

**Published:** 2022-10-16

**Authors:** Bettina Strommer, Dietmar Schulze, Bernhard Schartel, Martin Böhning

**Affiliations:** Bundesanstalt für Materialforschung und -Prüfung (BAM), Unter den Eichen 87, 12205 Berlin, Germany

**Keywords:** elastomers, graphene, crosslinking, network, rubber, vulcanization, nanocomposite

## Abstract

Tailored crosslinking in elastomers is crucial for their technical applications. The incorporation of nanoparticles with high surface-to-volume ratios not only leads to the formation of physical networks and influences the ultimate performance of nanocomposites, but it also affects the chemical crosslinking reactions. The influence of few-layer graphene (FLG) on the crosslinking behavior of natural rubber is investigated. Four different curing systems, two sulfur-based with different accelerator-to-sulfur ratios, and two peroxide-based with different peroxide concentrations, are combined with different FLG contents. Using differential scanning calorimetry (DSC), vulcametry (MDR) and swelling measurements, the results show an accelerating effect of FLG on the kinetics of the sulfur-based curing systems, with an exothermic reaction peak in DSC shifted to lower temperatures and lower scorch and curing times in the MDR. While a higher accelerator-to-sulfur ratio in combination with FLG leads to reduced crosslinking densities, the peroxide crosslinkers are hardly affected by the presence of FLG. The good agreement of crosslink densities obtained from the swelling behavior confirms the suitability of vulcameter measurements for monitoring the complex vulcanization process of such nanocomposite systems in a simple and efficient way. The reinforcing effect of FLG shows the highest relative improvements in weakly crosslinked nanocomposites.

## 1. Introduction

The wide use of elastomers today, from tires of all kinds, sealings for hydrogen storage, to very thin elastic membranes, would have been impossible with the sticky, unstable material Charles Goodyear experimented with almost 200 years ago. With the discovery of the vulcanization process, this sticky material became stable, elastic, and durable; adding sulfur and heating the material crosslinks the polymeric chains [1]. Today there are even more methods to vulcanize elastomers, and far more elastomers than only natural rubber. Depending on the application and the desired properties, curing systems based on sulfur, peroxide, or bifunctional molecules are used to achieve certain degrees of crosslinking [1,2,3]. Various other agents, such as activators, accelerators, and retarders, help to adjust the optimum scorch time (time until the crosslinking reaction starts) and the optimum curing time at a certain temperature in sulfur-based curing systems, in order to avoid long processing times and therefore possible degradation phenomena. They also ensure process safety and prevent premature crosslinking, i.e., in the injection molding unit [4]. These agents also influence the nature of the crosslinking; for example, a high sulfur-to-accelerator ratio promotes the formation of polysulfide chains connecting the polymer chains (conventional sulfur crosslinking). Conversely, a relatively higher amount of accelerator compared to the sulfur added leads to mono- and disulfide connections (efficient sulfur crosslinking) [5,6]. Peroxides, on the other hand, enable radical-based crosslinking between the polymer chains themselves [7,8].

The combination of elastomers and nanoparticles has been a success story for over 100 years; the incorporation of carbon black greatly improved the mechanical properties of natural rubber. The development of novel nanoparticles as reinforcing fillers in recent years, such as graphene, carbon nanotubes, and nano silicate, enabled an even broader spectrum of applications and greatly enhanced the material performance of elastomers. Especially the simultaneous significant reduction of conventional filler contents allowed for the more targeted optimization of property profiles [9,10,11,12,13,14,15,16,17,18,19,20]. Generally, nanoparticles facilitate the improvement of various properties, such as mechanical (i.e., increase of Young’s modulus), flame retardancy, as well as electrical and thermal conductivity [14,15,17,18,20,21,22]. Substantial science and research efforts have been dedicated to studying the influencing factors in order to fully exploit the reinforcing potential and to develop effective production routes for nanocomposites [22,23,24,25]. In this context, the curing behavior and the crosslinking characteristics are crucial for the technical properties of rubber products as well. When developing elastomeric nanocomposites, some influence of nanoparticles on the crosslinking reaction, its kinetics, and the resulting network is to be expected, as reported in [26,27,28]. However, the direction of this influence differs in a range that is as broad as the variety of nanocomposites. Depending on the type of elastomer and nanoparticles as well as their functionalization, shape, filler content, and chemical composition, and depending on the curing system and its additives and agents, crosslinking can be either delayed, prevented, accelerated, or enhanced [11,21,29,30,31,32,33,34,35,36,37,38,39,40]. Xing et al. developed a method using only graphene oxide for radical crosslinking [41]. The combination of nanoparticles and elastomers is more than just the summation of the two components; the cause and explanation of the effects on crosslinking demands intense investigation and for selected parameters to be systematically varied while others are kept fixed. Unfortunately, the current literature gives no profound answers to most of the open questions. To address this issue, different crosslinking systems should be combined with different nanoparticle contents with identical types of nanoparticles and elastomers.

In this study, the impact of commercially available few-layer graphene (FLG) with a stack thickness of around 12 atomic layers was incorporated in natural rubber via a latex premixing masterbatch route at five different filler contents (0, 1, 3, 5, and 10 parts per hundred rubber (phr)). Although the exact manufacturing process of the FLG is unknown, an oxidation step and a thermally reduction step are assumed to be required. The investigated curing systems used to vulcanize the nanocomposites were two sulfur-based systems with different accelerator-to-sulfur ratios (conventional or efficient), and two peroxide systems with different peroxide contents (1 phr and 3 phr peroxide). The efficient sulfur and 1 phr peroxide crosslinked samples represent relatively low degrees of crosslinking, and the conventional sulfur and 3 phr peroxide samples relatively high degrees of crosslinking. With the aid of differential scanning calorimetry and a moving die rheometer, the vulcanization kinetics were studied. Swelling experiments complemented by density measurements allowed the resulting crosslink density in the vulcanized nanocomposites to be determined in accordance with Flory–Rehner. The aim was to reveal the influence of the FLG and its concentration in the nanocomposite on the different curing systems and degrees of crosslinking. The mechanical property Shore A hardness provides first insights into the performance of the nanocomposites. The results help us to understand the reinforcing mechanisms of graphene in elastomers and raise awareness of the great impact of nanoparticles, not only to improve the final properties of nanocomposites, but also to avoid material degradation during processing or premature vulcanization. Finally, the study seeks to assess curing systems with regard to how they support the reinforcement effect of graphene in order to allow a more efficient comparison of the existing literature on graphene/rubber nanocomposites and the improvements achieved. The understanding of the influencing mechanisms of FLG on the crosslinking reaction and the resulting network structure not only allows for the more efficient development of nanocomposites, but it is also a prerequisite for the practical implementation of this approach.

## 2. Materials and Methods

### 2.1. Materials and Preparation

The nanocomposite preparation was a multi-step process consisting of masterbatch premixing, compounding, and vulcanization:

- Masterbatch premixing: 30 g of few-layer graphene (FLG, available as EXG R 98 300 from Graphit Kropfmühl GmbH, Untergriesbach, Germany) with a specific surface area (Brunauer–Emmett–Teller (BET)) of 326 m^2^ g^−1^ was introduced into distilled water at a concentration of 10 g L^−1^, followed by 5 min of mechanical stirring (stirring unit Ministar 20 digital with anchor stirrer R 1331, IKA^®^-Werke GmbH & CO.KG, Staufen, Germany) and an ultrasonication step of 1 h (UPS 400 S ultrasonicator with an H3 sonotrode, Hielscher Ultrasonics GmbH, Teltow, Germany). Then 100 g of natural rubber latex (high ammonia, solid content 60%, supplied by Weber & Schaer GmbH & Co. KG, Hamburg, Germany) was added while stirring. The rubber particles surrounding the FLG particles spontaneously started coagulating and thus initiated the formation of the solid masterbatch. Formic acid (5%) was added until the liquid became clear and a uniform solid masterbatch with a rubber-to-FLG ratio of 2:1 was formed (equivalent to 50 phr FLG content). The masterbatch was washed and rinsed until a neutral pH was obtained and put in a ventilating oven for 48 h at 50 °C for drying.
- Compounding: in a microcompounder (MC 15; Xplore Instruments BV, Sittard, The Netherlands) set to 100 °C and 75 rpm, 10 to 12 g of solid natural rubber (TSR-L from Dai Tieng Rubber Corporation, Dau Tieng, Vietnam) was masticated, followed by the addition of a certain amount of masterbatch, depending on the required FLG content in the final nanocomposite (0, 1, 3, 5, and 10 phr FLG). After dispersing and distributing the FLG masterbatch, the curing agents were added and dispersed, depending on the vulcanization system. The exact recipes (Table S1) and mixing protocols (Table S2) are given in the Supplementary Materials. The sulfur-based recipes were selected according to the suppliers’ recommendations [42]. Sulfur with an oil content of 5% was supplied by CS Additive GmbH, Germany, and the activator stearic acid (BAEROCID SP-1 A) from Baerlocher GmbH, Unterschleißheim, Germany. The accelerators n-cyclohexyl-2-benzothiazolesulfenamide (CZ, available as Vulkazit CZ/EG-C) and the activator zinc oxide (Zinkoxyd aktiv) were purchased from Lanxess Deutschland GmbH, Germany. Tetramethylthiuram disulfide (TMTD, available as Dimacit TMTD-PDR-D) was purchased from Taminco N.V., Ghent, Belgium. Dicumyl peroxide (Perkadox BC-FF) was supplied from Akzo Nobel Functional Chemicals B.V, Amsterdam, Netherlands. Carbon black (CB, Corax^®^ N 330) was bought from Orion Engineered Carbons GmbH, Senningerberg, Grand Duchy of Luxembourg.
- Vulcanization: disc-shaped specimens with diameters of 36.6 mm and thicknesses of 2 mm were vulcanized in a hot press at 150 °C (SulCon and SulEV) or at 170 °C (1Perox and 3Perox) and 300 bar pressure. For high material efficiency, respective molds were used. The vulcanization times in the hot press were set according to the optimum curing times t_c90_ obtained from vulcameter measurements (presented in Section 3.2) and differed for each curing system and FLG content.

### 2.2. Characterization Methods

To study the crosslinking reaction, differential scanning calorimetry (DSC) measurements were conducted using a DSC 204 F1 “Phoenix” (NETZSCH Instruments, Selb, Germany). For each material, three samples of ca. 10 mg were taken after compounding and placed in aluminum pans sealed with pierced lids; the heat flux was measured in a temperature ramp from 30 °C to 260 °C using a heating rate of 10 K min^−1^ in nitrogen atmosphere (N_2_ flow of 40 mL min^−1^). As the scattering was high, especially in the calculated enthalpies, for most samples the measurements were repeated twice. The results discussed in Section 3.1 therefore represent the respective mean values and standard deviations.

In a vulcameter (moving die rheometer (MDR), D-MDR 3000, Montech Werkstoffprüfmaschinen GmbH, Buchen, Germany), samples of 5 g unvulcanized compound were characterized in terms of their time-dependent crosslinking behavior. The curing behavior was studied in shear mode with a frequency of 1.66 Hz and an amplitude of 0.5°. All SulCon and SulEV samples were measured at 150 °C, and 1Perox and 3Perox samples at 170 °C. The minimum (ML) and maximum (MH) torque, the scorch time (t_s2_), and the optimum curing time (t_c90_) were determined according to ASTM D2084-19a.

Sorption and swelling measurements were conducted on three vulcanized, disc-shaped specimens (diameter of 36.6 mm, thickness of 2 mm) in isooctane (2,2,4-trimethylpentane, analytical grade, Chemsolute, Th. Geyer GmbH & Co. KG, Renningen, Germany) at room temperature. The specimens were immersed in the solvent, and changes in their mass were measured at certain intervals by removing them from the solvent batch, swabbing their surface, and weighing them in a weighing bottle on an analytical balance with a readability of 0.1 mg. The specimens were immediately returned to the solvent bath after weighing. Their density was measured in the dry state according to DIN EN ISO 1183-1. With the results from swelling and density measurements, crosslinking densities were calculated with the Flory–Rehner equation [43]:

(1) $$\upsilon_{cross}\left({\mathrm{mol}\;g}^{-1}\right)=-\frac{\ln\left(1-\upsilon_{r}\right)+\upsilon_{r}+\chi \upsilon_{r}^{2}}{2\rho_{r}V_{s}\left(\sqrt[{3}]{\upsilon_{r}}-\frac{\upsilon_{r}}{2}\right)},$$

$\upsilon_{cross}$ crosslinking density according to Flory–Rehner;

$\upsilon_{r}$ volume fraction of the equilibrium swollen rubber;

χ Flory–Huggings polymer solvent interaction parameter (0.49) [44];

$\rho_{r}$ density of rubber;

$V_{s}$ molar volume of the solvent (165.1 cm^3^ mol^−1^ for isooctane).

Detailed information about this calculation and the values used are given in Section 2 and Table S5 of the Supplementary Material.

Shore A hardness measurements were conducted according to ISO 48-4 on a Digi Test II instrument on three stacked samples (diameter of 36.6 mm and thickness of 2 mm), with a resulting thickness of 6 mm (Bareiss Prüfgerätebau GmbH, Oberdischingen, Germany). The obtained hardness value was calculated as the mean value of 9 measurements.

## 3. Results

### 3.1. Differential Scanning Calorimetry

**Figure 1:**
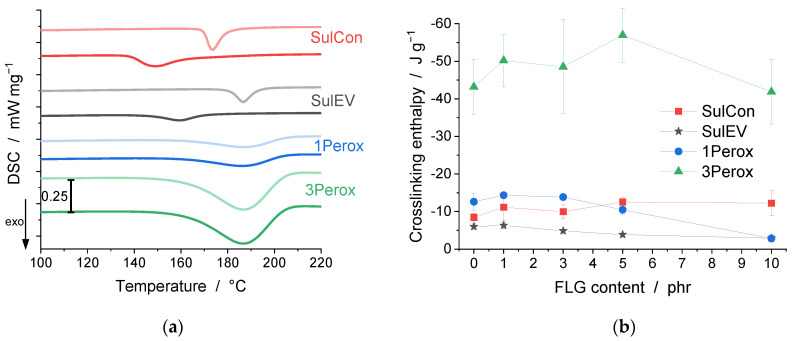

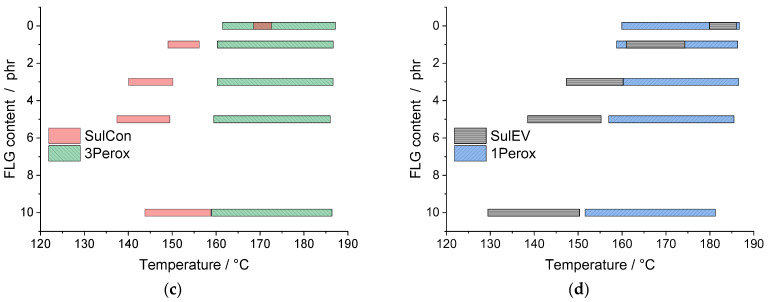
Representative heat flux (DSC) curves for 0 phr FLG (compact lines) and 3 phr FLG (dotted lines) for all curing systems are shown in (**a**), and exothermal crosslinking enthalpy (integral under the curves) over FLG content in (**b**). Diagrams (**c**,**d**) visualize the onset to peak temperature ranges (approx. half of the exothermal reaction peak ranges) for all FLG contents of SulCon and 3Perox, and SulEV and 1Perox, respectively.

To gain insight into the kinetics of the crosslinking reaction in the studied nanocomposites, DSC measurements were conducted. The exothermic heat flux indicated the starting temperature of the complex crosslinking reaction by means of the onset temperature, its maximum at the peak temperature, and the area under the heat flux curve, which is attributed to the reaction enthalpy. Table S3 in the Supplementary Material lists the values of the onset and peak temperatures, and the crosslinking enthalpies of the DSC measurements. Figure 1a shows representative DSC curves of unfilled NR for all investigated curing systems (compact lines), and in comparison, for nanocomposites with a FLG content of 3 phr (dotted lines). For the sulfur-based curing systems, SulCon and SulEV, the presence of FLG shifted the exothermic peak of the onset and peak temperatures to lower values. The shape of the peak broadened significantly, and therefore the distance between the onset and peak temperature increased. To further illustrate this broadening of the peaks for the curing systems in the studied FLG contents, Figure 1c,d visualize this distance between onset and peak temperature as bars that are approximately half of the peak width. For SulCon, the presence of 1 phr FLG led to a shift and broadening of the peak, which was further extended up to a content of 5 phr FLG. A value of 10 phr FLG, however, showed a tendency to shift to higher temperatures. SulEV nanocomposites showed the same trend of shifting the reaction peaks to lower temperatures and an even more pronounced broadening. In this case, 10 phr FLG behaved consistently as well. The reaction peaks of the peroxide-cured nanocomposites remained in similar ranges for all FLG contents and both peroxide contents, 1 phr in 1Perox and 3 phr in 3Perox, and showed no tendency to broaden. The observed shift to lower onset temperatures indicated an accelerating effect of FLG on the sulfur-based crosslinking. The crosslinking dynamics of SulEV was more affected by FLG than that of SulCon. Because of the higher accelerator-to-sulfur ratio of SulEV, a greater effect on the CZ and TMTD accelerators was expected than on the crosslinking with the sulfur atoms itself. The broadening of the respective DSC peaks may indicate a higher viscosity or a barrier effect of the FLG particles. The FLG can prevent the migration of the agents in the compound, which leads to prolonged reaction times overall, even though the start of the reaction occurred earlier. In the peroxide systems only one single agent, the cleaving peroxide, leads to crosslinking and is therefore less affected by a higher viscosity or a potential barrier effect of FLG. The enthalpy as the integral under the DSC peak showed great deviation, as demonstrated in Figure 1b. Especially for the 3Perox system, the enthalpies scattered over a broad range. This scattering may have resulted from inhomogeneities in the material. In comparison to the sulfur-based agents, the peroxide crosslinking led to higher enthalpies overall. In the peroxide crosslinked samples, no trend was observed, which could indicate whether FLG formed additional covalent crosslinks or prevented crosslinking reactions. The standard deviation was very high, especially for 3Perox. The 1Perox 10 phr FLG resulted in a very low crosslinking enthalpy, which was explained by its processing in the microcompounder used to compound all nanocomposites. For the 1Perox 10 phr FLG sample, a strong increase in torque was detected, until the engine was stopped automatically to avoid overheating. This rise in torque was an indication of premature crosslinking, and therefore the crosslinking enthalpy in the DSC showed only the crosslinking that took place after processing. The crosslinking enthalpy of SulCon showed a slight tendency to increase along with FLG content; see Figure 1b. In contrast, increasing the content of FLG clearly decreased the crosslinking enthalpy of SulEV, resulting in a crosslinking enthalpy reduction by 50% (−6.0 J g^−1^ for SulEV 0 phr FLG as compared to −2.9 J g^−1^ for SulEV 10 phr FLG).

### 3.2. Vulcametry/Moving Die Rheometer

**Figure 2:**
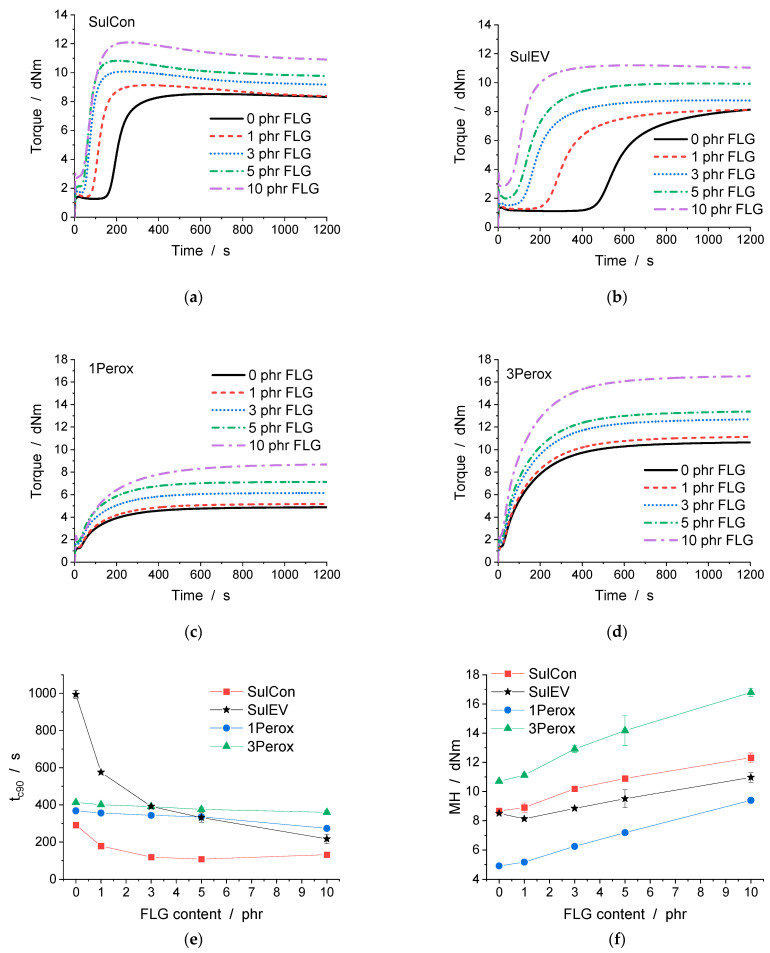
The vulcanization curves from MDR as torque over time are shown for (**a**) SulCon, (**b**) SulEV, (**c**) 1Perox, and (**d**) 3Perox with all FLG contents. Optimum curing time t_c90_ (**e**) and maximum torque MH (**f**) are displayed over FLG content.

A moving die rheometer (MDR) used as a vulcameter gives direct insight into the crosslinking kinetics and viscosity of the uncured compound and provides indications of the final product’s mechanical behavior. Via the isothermal measurement of the torque required to apply a certain strain at a certain frequency and temperature, this special plate-plate-rheometer setup applies a constant strain over the radius of the disc-shaped specimens. Grooves in the top and bottom of the rheometer chamber prevent slippage. A typical curing curve of elastomers starts with the sample reaching the set temperature (chosen depending on the type of rubber and curing system) and therefore an initial decrease in viscosity. This leads to a drop in torque until the minimum torque (ML) is reached. The increased temperature starts off the crosslinking reaction, and the torque begins to rise. As an indicator for the time when the crosslinking reactions set in effectively, the scorch time (t_s2_) describes the time until a torque of ML + 2 units (i.e., dNm) is attained. The torque rises until a plateau is reached, in some cases followed by a slight further increase or drop (due to incipient decomposition). The maximum torque (MH) is an indication of the elastomer’s final mechanical properties; the difference ΔS between MH and ML is correlated to the degree of crosslinking. The optimum curing time (t_c90_), defined as the time until 90% of ΔS is reached, is set as the curing time for production. Figure 2 shows representative curing curves for SulCon in Figure 2a, SulEV in Figure 2b, 1Perox in Figure 2c, and 3Perox in Figure 2d, respectively. SulCon nanocomposites had a more extended induction period before the torque started to rise. The torque of 3Perox nanocomposites rose nearly immediately, indicating that the crosslinking started as soon as the respective set temperature was reached in the sample. For all systems, FLG increased the torque levels even before vulcanization started and after reaching the plateau of MH, leading to torque levels of +18% (SulCon 3 phr FLG) and +42% (SulCon 10 phr FLG) MH compared to unfilled SulCon, and +21% (3Perox 3 phr FLG) and +57% (3Perox 10 phr FLG) MH compared to unfilled 3Perox. Table S4 in the Supplementary Material lists all results from the MDR measurements in detail. Similar to the shift in onset and peak temperature in DSC, FLG affected the kinetics represented for the vulcameter by t_s2_ and t_c90_ nanocomposites with sulfur-based curing: 3 phr of FLG decreased t_s2_ by 65% in SulCon and by 73% in SulEV. The addition of more FLG shortened the scorch times even further. The scorch times of nanocomposites containing peroxide also decreased with increasing FLG content, but in a less pronounced manner; see Table S4. The optimum curing time t_c90_ followed the same trends.

### 3.3. Sorption and Swelling Measurements

**Figure 3:**
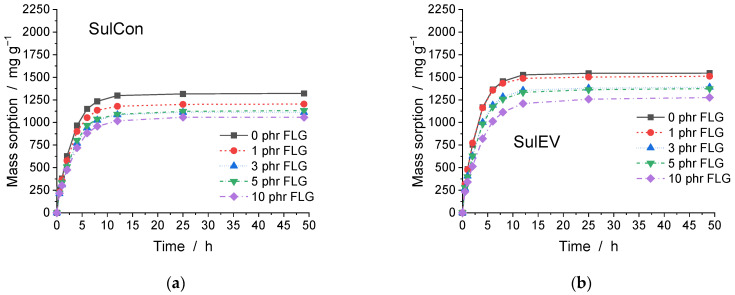

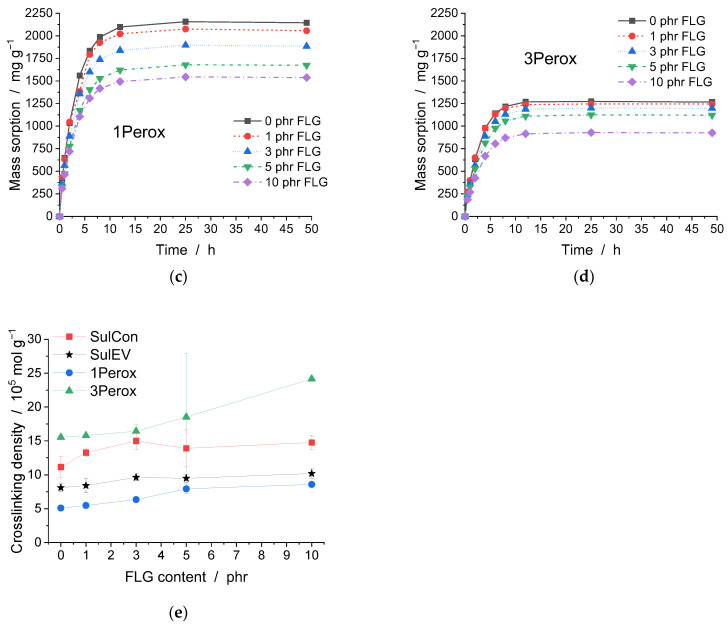
Development of the mass sorption over time shown for (**a**) SulCon, (**b**) SulEV, (**c**) 1Perox, and (**d**) 3Perox; (**e**) shows the crosslinking density over FLG content.

Measuring the ability of an elastomer to take up solvent is an efficient method to evaluate various material properties. The mass sorption is an important technical parameter for pipe and sealing applications, as the time dependency of this process gives insight into the permeation and diffusion behavior of the material. The covalent polymeric network as well as the reinforcing FLG prevent an extension of the material, comparable to an internal cage. This blockade prevented further mass uptake, resulting in a higher reduction of the equilibrium sorption than only the replacement of swellable rubber would have caused. The equilibrium volume swelling in combination with density measurements allows the crosslinking density υ_cross_ to be calculated in accordance with Flory–Rehner (see Equation (1)). Figure 3 shows the mass sorption over time for all final nanocomposites crosslinking with SulCon (Figure 3a), SulEV (Figure 3b), 1Perox (Figure 3c), and 3Perox (Figure 3d). Table S5 in the Supplementary Material lists the equilibrium sorption (after 49 h) and the calculated υ_cross_ with all parameters necessary for calculating the sorption values for the studied nanocomposites. The choice of curing system was the main factor influencing the sorption behavior of the investigated natural rubber nanocomposites. The general level of sorption was set by the curing system, although the FLG content further changed the mass uptake. For example, unfilled SulCon (0 phr FLG) showed an equilibrium sorption of 1322 mg g^−1^, which was in the same range as the SulEV nanocomposite with 10 phr FLG (1276 mg g^−1^). This effect was even more pronounced in the peroxide nanocomposites: 1Perox 10 phr FLG took up 1537 mg g^−1^, and the mass increase of 3Perox 0 phrFLG was 1267 mg g^−1^. Hence, the same trends were detected in the crosslinking densities; see Figure 3e. The υ_cross_ determined based on mass uptake measurements represents a combination of covalent crosslinking points due to chemical crosslinking and a physical network formed by FLG interacting with itself and the surrounding polymeric matrix. The calculated υ_cross_ of 3Perox was three times higher than the υ_cross_ of 1Perox, indicating a linear increase in crosslinks with increasing peroxide content, at least in the range of peroxide additive studied here. With regard to the sulfur-crosslinked nanocomposites, the addition of FLG led to a greater increase of υ_cross_ in SulCon than in SulEV; 3 phr of FLG led to a rise of 34% in SulCon, compared to 0 phr FLG. In SulEV, the same addition of 3 phr FLG increased υ_cross_ by only 19%.

The presence of FLG barely affected the sorption dynamics. Figure S1 in the Supplementary Material shows the normalized sorption curves (normalized to the equilibrium sorption of 1.0) over the square root of time. As the differences in the nanocomposites were very small, no significant effect of FLG on the sorption kinetics was detected. Furthermore, the largely linear behavior in these diagrams indicated Fickian diffusion behavior.

### 3.4. Hardness

**Figure 4:**
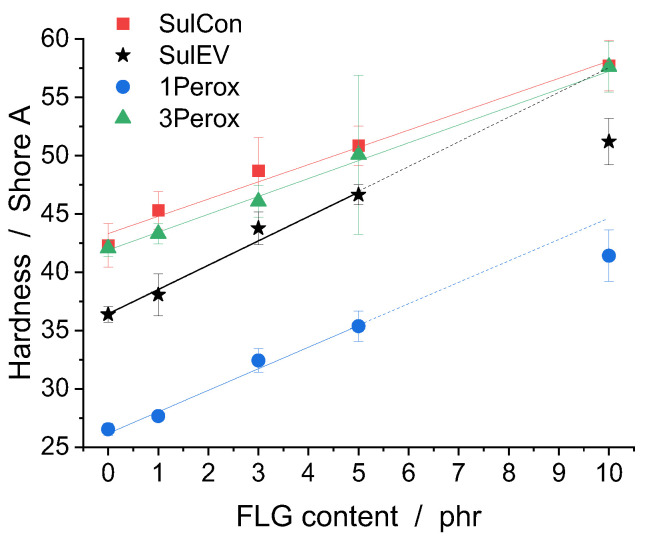
Shore A hardness for all curing systems over FLG content.

Hardness is the resistance of a material against mechanical indentation or penetration. Apart from several method-dependent factors, the hardness in elastomers is coupled with the Young’s modulus and the viscoelastic properties of the elastomer [45]. Figure 4 visualizes the Shore A hardness of the studied nanocomposites over the FLG content. The hardness increased linearly with FLG content for all studied curing systems. The parameters of the linear fits and their R^2^ values are listed in Table S6 along with the Shore A hardness values. The two highly crosslinked systems, SulCon and 3Perox, had similar hardness levels for every FLG content, and their fit parameters were in a similar range. Compared to that, SulEV and 1Perox, with lower crosslinking densities, differed greatly in terms of their hardness values with increasing FLG content. When the hardness values of SulEV 10 phr FLG and 1Perox 10 phr FLG, respectively, were included into the linear fit, R^2^ was lower for SulEV and 1Perox, indicating that this measurement point did not follow the same trends as the hardness values of 0 phr to 5 phr FLG. Therefore, for SulEV and 1Perox, the linear relation was conducted for the FLG contents of 0 to 5 phr, and the dotted lines in Figure 4 are for visualization only. The 10 phr FLG samples of SulEV and 1Perox showed lower hardness than predicted from the nanocomposites with lower FLG contents. This indicates that with further increases in FLG addition the increase in hardness seemingly started to level off. The slopes of the fits of SulEV and 1Perox were almost equal and higher than in SulCon and 3Perox, as listed in the Supplementary Material in Table S6.

## 4. Discussion

**Figure 5:**
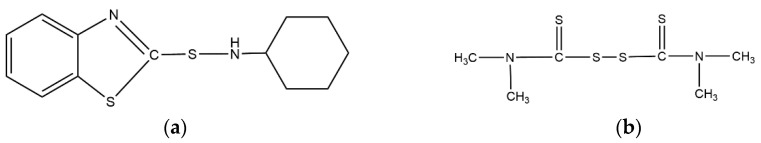
Chemical compositions of the accelerators n-cyclohexyl-2-benzothiazolesulfenamide CZ (**a**) and tetramethylthiuram disulfide TMTD (**b**).

Graphene as a nanoscaled filler distinctly influences the vulcanization behavior of NR. The results demonstrate an accelerating effect of FLG on the kinetics of sulfur crosslinking, with a strong indication that the reactions of the accelerators CZ and TMTD (chemical structures in Figure 5) were enhanced. The accelerated sulfur crosslinking with activators and accelerators, as in this study, is a multi-step process [46,47]:

- Activator A and accelerator X form an A–X complex;
- The A–X complex reacts with sulfur S_8_ and forms an A–X–S_x_ complex;
- With the polymer chain R–H, a rubber-bound intermediate R–S_y_–X is formed;
- The accelerator X is replaced by another R–H, forming the initial polysulfide crosslinks R–S_x_–R;
- Crosslinking shortening, additional crosslinking, crosslinking deconstruction, S–S bond interchange, and main-chain modifications;
- Final vulcanizate network.

It was reported by Hosseini et al. that the incorporation of carbon black leads to an accelerating effect in the vulcanization of nanocomposites in styrene butadiene rubber [28]. The reported effect, however, was weaker, as a similar reduction of scorch time was obtained only at higher filler concentrations of carbon black compared to our results with FLG. Wu et al. suggested a possible reaction scheme of graphene/natural rubber nanocomposites with CZ accelerator; sulfur atoms might bond on functional groups on the surface of graphene (–COOH or –OH groups). The altered functional sulfide groups further react with accelerators and additional sulfur, leading to graphene being covalently bonded to the polymeric chains by these functional groups [26]. Mensah et al. studied the crosslinking dynamics of CZ-accelerated sulfur crosslinking in nitrile butadiene rubber (NBR) with graphene oxide (GO) and reduced graphene oxide (rGO) [36]. According to the hypothesis that the functional oxygen groups on the graphene surface play the major influential role, one would assume that GO led to a higher crosslinking density or accelerating effect. However, the greatest impact was found in the rGO nanocomposites. Among the nanocomposites studied here, FLG influenced the crosslinking kinetics of SulEV with higher CZ content the most. The results indicated that the effect was catalytic, such that graphene was not bonded covalently to the polymer chains. Using these methods, it was not possible to differentiate between chemical and physical networking points. Even though the kinetics of the CZ/TMTD accelerated nanocomposites shifted to lower temperatures and shorter times, FLG impeded crosslinking in SulEV, as apparent in the crosslinking enthalpy (DSC) and lower increase in ΔS and υ_cross_ with increasing FLG content compared to SulCon. This contradicts the assumption that FLG taking part in the chemical crosslinking is the major explanation for the studied acceleration effects. A reduction in accelerator content (CZ, TMTD, or others) might be possible for sulfur-based curing, but further investigations are necessary. As FLG provides a strong accelerating effect in sulfur-based crosslinking, peroxide systems bring advantages for sensitive processing steps, i.e., injection molding. The studied peroxide nanocomposites, 1Perox and 3Perox, showed a constant onset temperature and stable scorch times. In general, this study yielded no conclusive evidence for FLG being directly incorporated in the covalent, chemical crosslinking with the presented methods.

**Figure 6:**
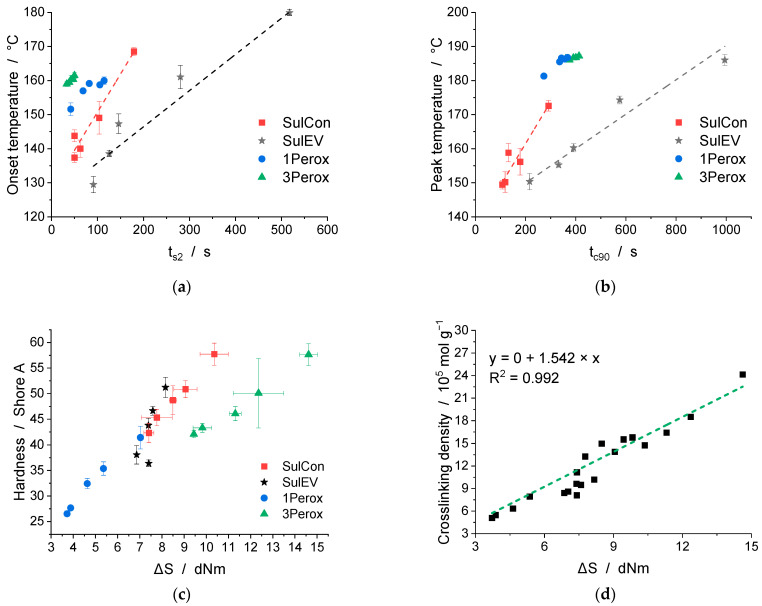
Correlations between the different methods and results: (**a**) onset temperature (DSC) over scorch time (MDR), (**b**) peak temperature (DSC) over optimum curing time (MDR), (**c**) Shore A hardness over ΔS (MDR), and (**d**) crosslinking density (swelling) over ΔS (MDR).

Figure 6 shows correlations between the results of DSC, MDR, swelling, and hardness measurements. As seen in Figure 6a,b the two characteristic temperatures from DSC, onset and peak temperature, correlated well with the scorch and optimum curing time from MDR, depending on the studied curing system and temperature set in the MDR (SulCon and SulEV: 150 °C, 1Perox and 3Perox: 170 °C). As a moving die rheometer (MDR) or rapid process analyzer (RPA) is often available in the elastomer processing industry and at research institutes, these results underline the reliable information this instrument provides for nanocomposites as well. Figure 6c,d demonstrate the correlation between ΔS and hardness, and ΔS and crosslinking density. The increase of torque in the MDR measurement reflects the tendency of the final nanocomposite’s mechanical properties, in this case the hardness. The determination of the crosslinking density υ_cross_ in accordance with Flory–Rehner requires a time-intensive measurement. ΔS correlates well with υ_cross_ via the shown linear relation. Therefore, MDR measurements are confirmed to be an efficient method to assess the crosslinking kinetics and the resulting network in such nanocomposite systems.

Apart from the crosslinking behavior, the results are important for technical applications. Since many studies have demonstrated that FLG or graphene content of up to 3 phr achieves high reinforcement, a comparison of the 3 phr FLG nanocomposites with their unfilled references (0 phr FLG) gives insight into the relative reinforcing effect; 1Perox provided the highest relative improvements through the addition of 3 phr FLG—hardness increased by 22% and MH by 27%, and mass sorption decreased by 13%. The denser network of 3Perox resulted in a lower reinforcing effect of 3 phr FLG, with +10% in hardness, +21% in MH, and −6% in mass sorption. The comparison of SulCon and SulEV showed more efficient reinforcing by 3 phr FLG in SulCon, namely, +15% in hardness, +17% in MH, and −16% in mass sorption for SulCon, compared to +20% in hardness, +5% in MH, and −10% in mass sorption for SulEV. While a lower crosslinking density seemed to result in higher relative reinforcement of 3 phr FLG, in SulEV the FLG’s effect on impeding the formation of crosslinking counteracted this effect.

The results give profound insights in the crosslinking behavior of natural rubber and FLG at different levels of filler content. However, for other matrices and nanoparticles, the drawn conclusions might not be directly transferable. Apart from different elastomers and other filler nanoparticles, the influence of the manufacturing or functionalization of graphene species such as FLG leaves room for future investigations in this study field.

## 5. Conclusions

Few-layer graphene affects the properties of elastomers. Apart from mechanical reinforcement, this study demonstrated the influence of the nanoparticle FLG on the crosslinking processes; sulfur-based crosslinking was accelerated, with a pronounced effect on formulations dominated by CZ/TMTD (efficient sulfur crosslinking). Peroxide systems showed more stable crosslinking kinetics in the presence of FLG. The resulting network in the nanocomposites consisted of a combination of a physical network and chemical, covalent crosslinking. With the presented methods (DSC, MDR, and swelling), no distinct indication of covalent bonds between natural rubber and FLG was obtained. However, the results demonstrated MDR to be an effective method for the investigation of the vulcanization behavior of nanocomposites. The hardness, maximum torque in MDR, and mass sorption in a solvent gave insight into the technical performance of the resulting nanocomposites and revealed the higher relative reinforcement potential of FLG for low-density crosslinked elastomers.

## Data Availability

Data can be made available upon request.

## Supplementary Materials

The following supporting information can be downloaded at: https://www.mdpi.com/article/10.3390/polym14204363/s1: Figure S1: normalized mass sorption over the square root of time, Table S1: Recipes of nanocomposites, Table S2: mixing protocols, Table S3: results of DSC measurements, Table S4: results of MDR measurements, Table S5: results of swelling and density measurements, and parameters for the determination of the crosslinking density according to Flory–Rehner, Table S6: results of Shore A hardness measurements, Equations (S1) and (S2): additional information on the calculation of the crosslinking density.
