# Supplementary material for: Networking Skills: The Effect of Graphene on the Crosslinking of Natural Rubber Nanocomposites with Sulfur and Peroxide Systems
Source: Polymers (Basel). 2022 Oct 16;14(20):4363. doi: 10.3390/polym14204363 (PMC9608841; doi:10.3390/polym14204363)
Supplement: Supplementary file 1 [file polymers-14-04363-s001.zip › polymers-1929720-supplementary.pdf]

polymers

MDPI

Supplemental Material

Networking Skills: The Effect of Graphene on the Crosslinking of Natural Rubber Nanocomposites with Sulfur and Peroxide Systems

Bettina Strommer, Dietmar Schulze, Bernhard Schartel and Martin Böhning *

Bundesanstalt für Materialforschung und -prüfung (BAM), Berlin
* Correspondence: martin.boehning@bam.de

1. Recipes and mixing protocols

Table S1. Recipes of nanocomposites (all amounts in phr)

	SulCon	SulEV	1Perox	3Perox
NR	100	100	100	100
Sulfur	2	0.5	-	-
CZ	0.7	3	-	-
TMTM	0.5	1	-	-
ZnO	5	5	-	-
Stearic acid	1	1	-	-
Peroxide	-	-	1	3
CB	0.5	0.5	0.5	0.5
FLG	0; 1; 3; 5; 10	0; 1; 3; 5; 10	0; 1; 3; 5; 10	0; 1; 3; 5; 10

Table S2. Mixing protocols of nanocomposites

	Timer / min	Addition	Comments
SulCon, SulEV	0 to 6	67% of total NR amount	Mastication
	7	FLG masterbatch	Mixing
	8	CZ, TMTM, ZnO, stearic acid, CB	Mixing
	9 to 13	Rest of NR	Mixing
	14	Sulfur	Mixing
	15		Cooling on
	17		Engine off
1Perox, 3Perox	0 to 4	67% of total NR amount	Mastication
	5	FLG masterbatch	Mixing
	6 to 7	90% of total NR amount	Mixing
	8	CB	Mixing
	9.5	Peroxide	Mixing
	10 to 13	Rest of NR	Mixing
	15		Cooling on
	17		Engine off

2. Crosslinking density according to Flory-Rehner

The following information provides further insight into the calculation of the crosslinking density (equation S1)

$$\upsilon_{cross}(mol\ g^{-1}) = -\frac{\ln(1-\upsilon_r)+\upsilon_r+\chi\upsilon_r^2}{2\rho_r V_S(\sqrt[3]{\upsilon_r}-\frac{\upsilon_r}{2})}, \quad (S1)$$

The volume fraction of the equilibrium swollen rubber υ_r was calculated according to equation S2:

$$\upsilon_r = \frac{\frac{w_0 - w_F}{\rho_{r0}}}{\frac{w_0 - w_F}{\rho_{r0}} + \frac{w_{eq} - w_0}{\rho_S}} \quad (S2)$$

where w_0 in g is the weight of the nanocomposite sample before the sorption measurement, w_{eq} is the weight of the same sample after reaching the equilibrium sorption mass, w_F is the weight of the filler (calculated by w_0 multiplied by the filler content in wt.-%, as the sum of ZnO, CB and FLG), ρ_{r0} is the density of the unfilled rubber sample (with 0 phr FLG content) and ρ_S is the density of the solvent isooctane (0.69 g cm^{-3}). X is the Flory-Huggings parameter, at 0.49 for isooctane and natural rubber.

3. Differential Scanning Calorimetry

Table S3. Results of DSC measurements

Curing system	FLG content / phr	Crosslinking enthalpy / J g^{-1}	Onset temperature / °C	Peak temperature / °C
SulCon	0	-8.5 ± 1.0	168.5 ± 1.2	172.6 ± 1.6
	1	-11.1 ± 3.5	149.1 ± 4.8	156.2 ± 3.9
	3	-9.9 ± 1.9	140.0 ± 2.5	150.2 ± 3.0
	5	-12.5 ± 1.2	137.4 ± 1.4	149.5 ± 1.3
	10	-12.2 ± 3.3	143.8 ± 1.7	158.8 ± 2.7
SulEV	0	-6.0 ± 0.7	179.9 ± 0.9	186 ± 1.6
	1	-6.3 ± 0.7	161.0 ± 3.4	174.3 ± 1.2
	3	-4.9 ± 0.4	147.3 ± 2.9	160.3 ± 1.2
	5	-3.9 ± 0.5	138.5 ± 0.9	155.2 ± 0.7
	10	-2.9 ± 0.5	129.5 ± 2.4	150.3 ± 2.4
1Perox	0	-12.6 ± 2.2	159.9 ± 1.1	186.8 ± 0.4
	1	-14.3 ± 0.5	158.7 ± 0.4	186.3 ± 0.6
	3	-13.8 ± 0.1	159.1 ± 0.7	186.6 ± 0.7
	5	-10.5 ± 1.2	157.0 ± 0.7	185.5 ± 0.5
	10	-2.8 ± 0.2	151.6 ± 1.8	181.3 ± 0.1
3Perox	0	-43.2 ± 7.4	161.5 ± 0.8	187.2 ± 0.5
	1	-50.2 ± 6.9	160.3 ± 0.7	186.7 ± 0.4
	3	-48.5 ± 12.5	160.3 ± 1.3	186.7 ± 0.3
	5	-57.0 ± 7.2	159.5 ± 0.6	186 ± 0.1
	10	-41.9 ± 8.6	159.0 ± 0.7	186.4 ± 0.3

4. Vulcametry / Moving Die Rheometer

Table S4. Results of MDR measurements

Curing system	FLG content / phr	MH / dNm	ML / dNm	Δ S / dNm	t_{s2} / s	t_{c90} / s
SulCon	0	8.7 ± 0.2	1.3 ± 0.1	7.4 ± 0.2	179.1 ± 4	291 ± 8
	1	8.9 ± 0.3	1.1 ± 0.4	7.8 ± 0.7	103.5 ± 7	178 ± 6
	3	10.2 ± 0.2	1.7 ± 0.1	8.5 ± 0.2	62.4 ± 3	119 ± 3
	5	10.9 ± 0.1	1.8 ± 0.5	9.1 ± 0.5	49.8 ± 4	108 ± 1
	10	12.3 ± 0.3	2.0 ± 0.3	10.4 ± 0.6	49.8 ± 4	132 ± 4
SulEV	0	8.5 ± 0.1	1.1 ± 0.1	7.4 ± 0.1	517.5 ± 11	994 ± 20
	1	8.1 ± 0.1	1.3 ± 0.1	6.9 ± 0.1	280.2 ± 1	575 ± 1
	3	8.9 ± 0.1	1.5 ± 0.1	7.4 ± 0.2	146.1 ± 17	392 ± 8
	5	9.5 ± 0.7	1.9 ± 0.1	7.6 ± 0.7	125.6 ± 1	332 ± 25
	10	11.0 ± 0.3	2.8 ± 0.1	8.2 ± 0.4	91.1 ± 2	216 ± 26
1Perox	0	4.9 ± 0.1	1.2 ± 0.1	3.7 ± 0.1	114.4 ± 5	337 ± 5
	1	5.2 ± 0.1	1.3 ± 0.1	3.9 ± 0.1	105.1 ± 7	356 ± 2
	3	6.2 ± 0.1	1.6 ± 0.1	4.6 ± 0.2	82.0 ± 3	343 ± 1
	5	7.2 ± 0.1	1.8 ± 0.1	5.4 ± 0.1	68.6 ± 1	336 ± 2
	10	9.4 ± 0.1	2.4 ± 0.1	7.0 ± 0.1	41.9 ± 1	273 ± 1
3Perox	0	10.7 ± 0.1	1.3 ± 0.1	9.4 ± 0.1	49.8 ± 1	413 ± 1
	1	11.1 ± 0.1	1.3 ± 0.3	9.8± 0.4	47.1 ± 4	401 ± 1
	3	12.9 ± 0.2	1.6 ± 0.1	11.3 ± 0.3	42.3 ± 1	391 ± 3
	5	14.2 ± 1.0	1.8 ± 0.1	12.4 ± 1.1	38.4 ± 1	376 ± 10
	10	16.8 ± 0.3	2.2 ± 0.1	14.6 ± 0.4	33.0 ± 1	360 ± 12

5. Sorption and Swelling Measurements

Table S5. Results of swelling and density measurements, and parameters for the determination of the crosslinking density according to Flory-Rehner

Curing system	FLG content / phr	Density / g cm^{-3}	Filler content / wt.-%	υ_r / -	Equilibrium sorption / mg g^{-1}	Crosslinking density / 10^{-5} mol g^{-1}
SulCon	0	0.966	5.01	0.3396	1322 ± 75	12.4 ± 1.5
	1	0.973	5.87	0.3585	1203 ± 22	14.7 ± 0.5
	3	0.978	7.68	0.3722	1111 ± 44	16.6 ± 1.3
	5	0.986	9.15	0.3637	1132 ± 94	15.4 ± 2.7
	10	1.010	12.95	0.3706	1057 ± 73	16.4 ± 1.1
SulEV	0	0.960	4.99	0.3059	1544 ± 50	8.1 ± 0.6
	1	0.966	5.85	0.3083	1502 ± 90	8.4 ± 1.0
	3	0.974	7.51	0.3204	1379 ± 35	9.6 ± 0.5
	5	0.976	9.11	0.3183	1362 ± 37	9.5± 0.5
	10	1.003	12.90	0.3198	1257 ± 37	10.2 ± 0.7
1Perox	0	0.9176	0.49	0.2586	2157 ± 15	5.1 ± 0.1
	1	0.9202	1.46	0.2650	2075 ± 13	5.5 ± 0.1
	3	0.9295	3.41	0.2782	1897 ± 10	6.4 ± 0.1
	5	0.9384	5.16	0.2993	1680 ± 70	7.9 ± 0.7
	10	0.9489	9.42	0.3072	1545 ± 20	8.6 ± 0.2
3Perox	0	0.9198	0.48	0.3708	1273 ± 12	15.5 ± 0.3
	1	0.9250	1.44	0.3727	1245 ± 3	15.8 ± 0.1
	3	0.9345	3.29	0.3773	1199 ± 32	16.4 ± 1.0
	5	0.9451	5.07	0.3914	1125 ± 334	18.5 ± 9.4
	10	0.9687	9.25	0.4240	928 ± 1	24.1 ± 0.1

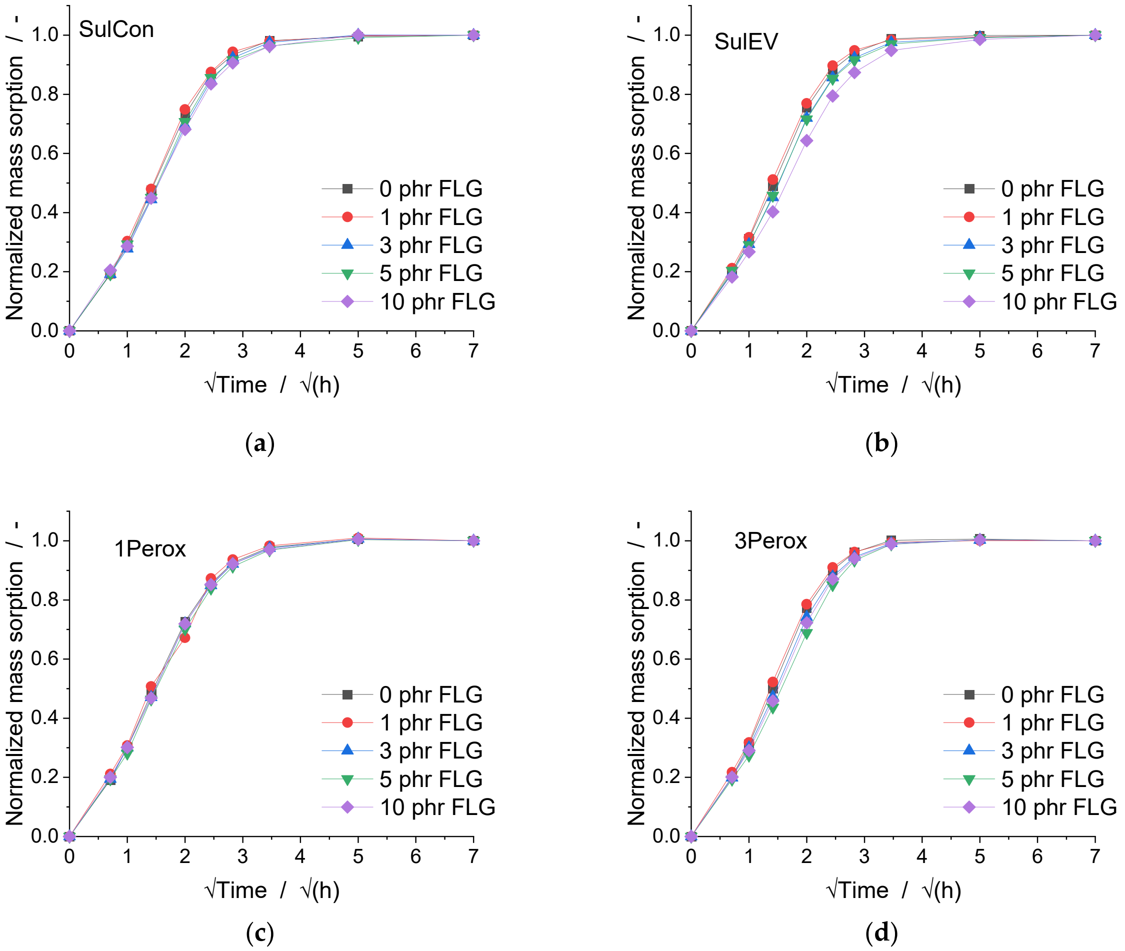

Figure S1. Normalized mass sorption over square root of time for SulCon (a), SulEV (b), 1Perox (c), and 3Perox (d)

6. Hardness

Table S6. Results of Shore A hardness measurements

Curing system	FLG content / phr	Hardness / Shore A	Linear fit in Figure 4.	R^2
SulCon	0	42 ± 2	y = 43.3 + 1.48 * x	0.984
	1	45 ± 2		
	3	49 ± 3		
	5	51 ± 2		
	10	58 ± 2		
SulEV	0	36 ± 1	y = 36.4 + 2.09 * x	0.992
	1	38 ± 2		
	3	44 ± 1		
	5	47 ± 1		
	10	51 ± 2		
1Perox	0	27 ± 1	y = 26.2 + 1.85 * x	0.973
	1	28 ± 1		
	3	32 ± 1		
	5	35 ± 1		
	10	41 ± 2		
3Perox	0	42 ± 1	y = 41.9 + 1.53 * x	0.996
	1	43 ± 1		
	3	46 ± 1		
	5	50 ± 7		
	10	58 ± 2		
